# Virtual Reality–Applied Home-Visit Rehabilitation for Patients With Chronic Pain: Protocol for Single-Arm Pre-Post Comparison Study

**DOI:** 10.2196/58734

**Published:** 2024-12-30

**Authors:** Hiroki Funao, Ryo Momosaki, Mayumi Tsujikawa, Eiji Kawamoto, Ryo Esumi, Motomu Shimaoka

**Affiliations:** 1 Department of Molecular Pathobiology and Cell Adhesion Biology Mie University Graduate School of Medicine Tsu Japan; 2 Department of Practical Nursing Mie University Graduate School of Medicine Tsu Japan; 3 Department of Rehabilitation Medicine Mie University Graduate School of Medicine Tsu Japan; 4 Faculty of Nursing Suzuka University of Medical Science Suzuka Japan; 5 Department of Intensive Care Medicine Mie University Hospital Tsu Japan; 6 Department of Emergency Medicine National Hospital Organization Mie Chuo Medical Center Tsu Japan

**Keywords:** chronic pain, homebound patient, home-visit rehabilitation, virtual reality, protocol, feasibility study, VR, pain, recurrent pain, home visit, rehabilitation, home rehabilitation, in-home, effective, screening, VR intervention, feasibility, alleviate pain

## Abstract

**Background:**

Pain inhibits rehabilitation. In rehabilitation at medical institutions, the usefulness of virtual reality (VR) has been reported in many cases to alleviate pain. In recent years, the demand for home rehabilitation has increased. Unlike in medical situations, the patients targeted for in-home rehabilitation often have chronic pain due to physical and psychosocial factors, and the environment is not specialized for rehabilitation. However, VR might be effective for in-home rehabilitation settings.

**Objective:**

This study aims to evaluate the feasibility of applying VR to home-visit rehabilitation for homebound patients with chronic pain.

**Methods:**

This study will test the feasibility of VR applied to home-visit rehabilitation for patients with chronic pain. A single-arm pre-post comparison will be conducted to evaluate its feasibility. Screening will be conducted on patients who have given consent to participate in the study, and those who have pain that persists or recurs for more than 3 months and receive home-visit rehabilitation will be enrolled in the study. Baseline measurements will be conducted on study participants before the start of the VR intervention. VR-applied home-visit rehabilitation will be conducted once a week for a total of 10 VR interventions. The primary endpoint is the change in pain from the baseline to the tenth intervention. Pain is a subjective symptom of the study participants and will be subjectively assessed by the Numerical Rating Scale of 11 levels from 0 to 10. Pain as the primary endpoint will be measured at 3-time points per rehabilitation session: before, during, and after the rehabilitation so that changes between time points can be evaluated. Secondary endpoints are heart rate variability, range of motion of the area in the musculoskeletal system where the pain occurs, motivation for rehabilitation, catastrophic thoughts of pain, mood state, quality of life, and interviews. Assessments will be conducted at the baseline, first, fifth, and tenth interventions. After completing the clinical study (10 VR interventions), patients will continue their regular home-visit rehabilitation as usual.

**Results:**

Recruitment of participants began on February 22, 2022, and data collection is ongoing as of November 2024. The research results will be published in international peer-reviewed journals and through presentations at national and international conferences.

**Conclusions:**

This study will contribute to the development of novel rehabilitation-based solutions for homebound patients who have had difficulty obtaining adequate relief from chronic pain. Future studies will consider conducting randomized controlled trials as clinical trials to validate the efficacy of VR during home-visit rehabilitation for patients with chronic pain.

**International Registered Report Identifier (IRRID):**

DERR1-10.2196/58734

## Introduction

Chronic pain is defined as pain that persists or recurs for longer than 3 months [1]. It is multifactorial with biological, psychological, and social factors and affects an estimated 20% of people worldwide [2]. Rehabilitation has been shown to be effective in improving not only functional disabilities but also chronic pain [3-5]. However, the increase in exercise-induced pain and the fear of exercise due to pain are challenges that hinder effective rehabilitation [6]. Furthermore, as indicated by the fear-avoidance model of pain [7], the experience of chronic pain caused by physical disabilities and exercise-induced pain associated with rehabilitation can increase anxiety and fear in patients. This leads patients to avoid physical activity, including rehabilitation, and the resulting inactivity and depression lead to a vicious cycle that exacerbates chronic pain.

Many homebound patients have chronic pain and functional disabilities [8]. These patients are often anxious and depressed due to restricted social participation [9]. Homebound patients have psychosocial factors, making it difficult to establish a treatment for chronic pain. As many homebound patients are unable to visit medical institutions, home rehabilitation is important. Unlike medical institutions, patients’ homes are not optimal environments for engaging in effective rehabilitation programs. As a result, patients find it difficult to engage in rehabilitation and are more likely to lose their motivation [10]. Maintaining motivation is more difficult when the home rehabilitation session is prolonged [11]. Home rehabilitation for patients with chronic pain requires an approach that not only relieves pain but also considers psychosocial factors, such as depression, anxiety, and low motivation.

Other chronic pain management measures include pharmacotherapy [12,13], interventional therapy [14], and psychotherapy [15]. However, the use of analgesics can lead to dependence, and, in older people with many underlying medical conditions, there is a risk of multiple drug use leading to adverse symptoms [16]. Furthermore, interventional treatment, which requires regular hospital visits and inpatient care, is burdensome for patients at home. Psychotherapy may also be difficult to manage adequately due to human and time constraints on visits by professional staff [17]. Therefore, there is a need for chronic pain management measures that are nonpharmacological and can be easily implemented at home.

Virtual reality (VR) is a 3D computer-generated simulated environment, which attempts to replicate real-world or imaginary environments and interactions [18]. In recent years, VR has been used in a variety of applications in the medical field, including to alleviate pain and facilitate rehabilitation. Immersion in a simulated virtual environment that produces powerful psychological effects, including relaxation, has been shown to be effective in relieving various types of pain, from acute to chronic [19,20]. VR brings a sense of presence that can be very overpowering and diminish a person’s ability to respond to noxious stimuli and attend to nociceptive neural signals, resulting in less perceived pain [21]. This mechanism is described as a distraction, and any strategy used to modulate pain in VR typically builds on a level of distraction [19]. A rehabilitation program with VR can help patients maintain a positive attitude toward rehabilitation and promote recovery of physical functions [22]. For in-home rehabilitation, a focus on motivation and medical costs for homebound patients has been reported to improve training effectiveness due to motivation [23,24]. However, there is currently insufficient clinical evidence regarding the effects of VR for chronic pain in home-visit rehabilitation. This study aims to evaluate the feasibility of applying VR to home rehabilitation for homebound patients with chronic pain. Alleviation of chronic pain using VR will help facilitate rehabilitation; thereby, rehabilitation will be more effective in improving chronic pain.

## Methods

### Overview

A single-arm pre-post comparison will be conducted to evaluate the feasibility. This study protocol has been developed using the findings from our case report on the VR applied to home-visit rehabilitation for hemiplegic shoulder pain in a patient with stroke [25]. In this case, VR was newly applied to a part of the home-visit rehabilitation that the patient had been working on. The VR-applied home-visit rehabilitation was carried out on a weekly basis, and during the intervention period, which was close to a month, the results suggested the chronic pain was continuously relieved compared with before the use of VR.

### Recruitment Process

The study participants will be recruited from several institutions. Screening will be conducted on patients who give consent to participate in the study, and those who meet the criteria will be enrolled as research participants.

Researchers contact the representative of the home health care agency providing the home rehabilitation service, explain the research outline, and establish a cooperative framework. Each representative picks up patients who meet the criteria. Researchers visit the patients, assess them for compliance with the criteria, and obtain explanations and consent. As the study period coincides with the COVID-19 pandemic, researchers should explain to patients and take the following actions: standard infection control measures should be taken during the researchers’ visit; research equipment should be controlled and disinfected by the researchers; and the possibility of COVID-19 infection in relevant personnel should be considered. If there is a possibility that the person concerned may be infected with COVID-19, the visit should be canceled or postponed.

### Inclusion Criteria

Patients who meet all of the criteria will be enrolled in the study and the criteria are to (1) have pain that persists or recurs for more than 3 months, (2) receive home-visit rehabilitation, (3) have adequate visual and auditory functions to use the VR equipment, (4) have adequate cognitive and perceptual functions to answer questions measuring subjective symptoms, (5) be at least 20 years of age at the time of consent, and (6) give their free and voluntary written consent to participate in the study, after being fully informed. As the study participants are patients with chronic pain in home care and may have different physical and cognitive ability levels, the following points should be noted; no sensory or cognitive impairment has been diagnosed by the family doctor; the patient is able to see and hear the content with the opportunity to trial the VR before the study begins; and there is a good level of understanding of the research description.

### Exclusion Criteria

Patients who meet any of the criteria will be excluded from the study and the criteria are to (1) have a history of adverse events due to the use of VR devices and (2) are deemed inappropriate for the study by the principal investigator.

### Sample Size

The sample size of 40 cases was not set by statistical detection. It was established based on the median number of cases in reports of studies using immersive VR for pain relief [26-29]. In this study, the targeted number of cases was set based on the assumption that due to the many older patients in the trial, there would be a dropout rate of approximately 15% during the intervention. This dropout rate was based on previous studies that used VR-based rehabilitation interventions [22,30].

### Information on Virtual Reality Devices and Contents

The intervention in this study is VR-applied home-visit rehabilitation. VR will be used during the rehabilitation procedures that the study participants usually engage in.

**Figure 1:**
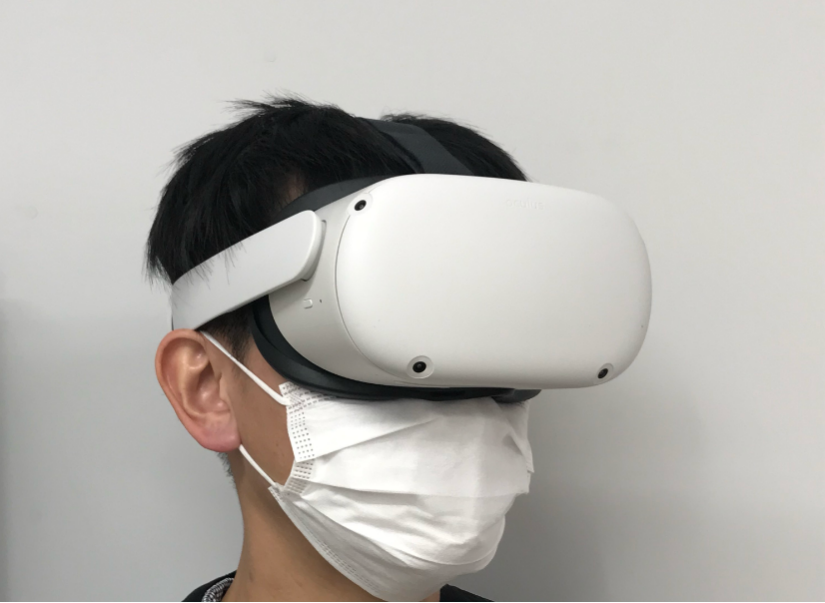
The virtual reality device which is used in this study (Meta quest2).

The VR device used in this study will be Meta Quest 2 (Meta Platforms Inc), which is one of the most widely used VR devices by the general public (Figure 1). Meta Quest is a headset-type VR viewing device, which is worn by fixing on the head a device covering the field of vision. It enables viewing of images in 360 degrees in all directions, in accordance with the frontal orientation of the face, and listening to environmental sounds that accompany the images. The researcher manages the VR device and brings the device to the patient’s home. If the patient has difficulty setting up and fitting the VR device themselves, the researcher will fit or assist them.

**Figure 2:**
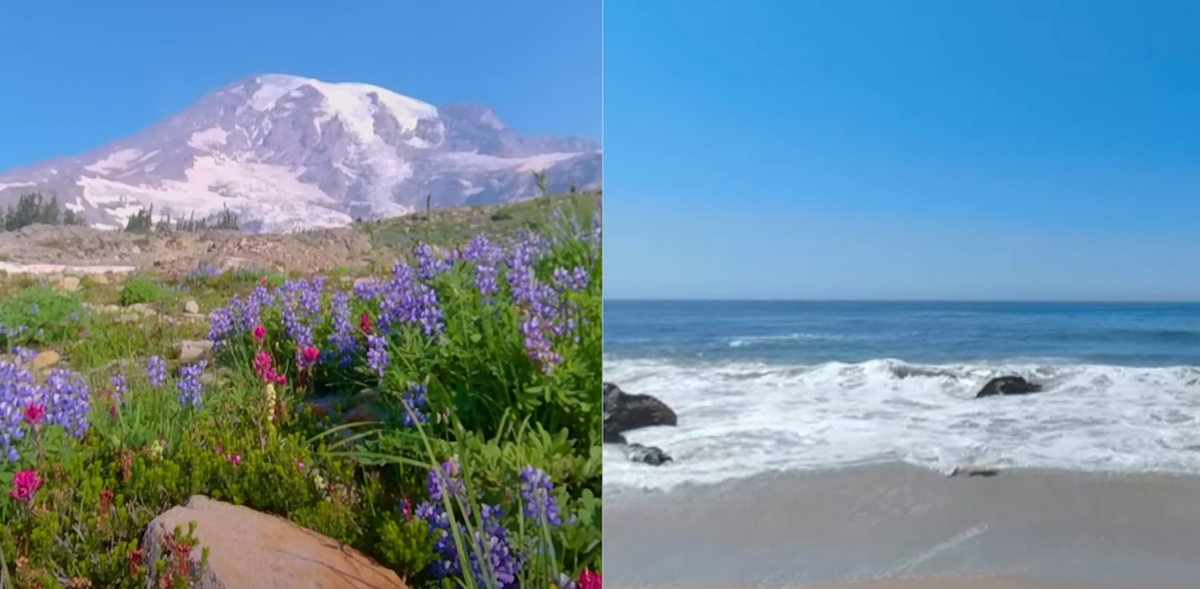
The virtual reality content is viewed with Meta quest2 (YouTube virtual reality).

The VR content viewed with Meta Quest 2 will be selected by researchers from natural landscapes available on YouTube VR [31]. This is because one of the VR contents that has the psychological effect of promoting pain relief is landscape [32,33]. In addition, to prevent adverse symptoms associated with the use of VR, the contents that do not change the viewpoint frequently and violently should be selected [34]. In addition, as the participants used VR during their usual rehabilitation, there is no need to perform online operations during VR viewing to avoid affecting the rehabilitation. These will include 10-15 minutes of content of landscapes such as beaches, flower fields, forests, and plateaus (Figure 2). The researcher is present when the VR is used and confirms, and records which content is viewed. The types of landscapes used will be changed regularly so that research participants can experience more than one type of content. The time of the home-visit rehabilitation will be about 40-60 minutes, and VR content will be provided during a part of that time. Rehabilitation will continue while the VR content is being experienced. To ensure the safety of the research participants, VR content will only be provided when they are in a supine or seated position, as wearing the VR equipment will block their full field of vision. Expected adverse events in this study will include pressure sores at the site where the VR equipment is worn, muscle strain from the neck to the shoulders (eg, pain, tightness, and heaviness) due to the load of the VR equipment, and discomfort from viewing VR images (VR sickness) [35], all of which are expected to be mild and tolerable. If any of these adverse events should occur, the VR will be stopped as soon as possible, the patient will be monitored, and medical attention will be sought if necessary.

### Data Collection

#### Outline

**Figure 3:**
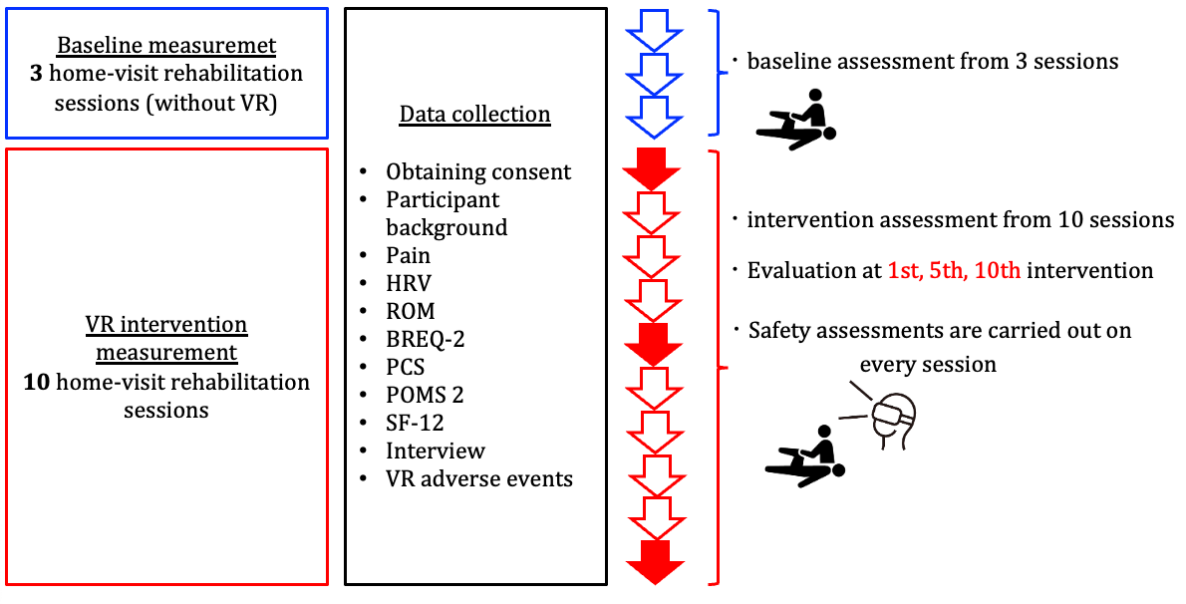
Outline for data collection. BREQ-2: Behavioral Regulation in Exercise Questionnaire-2; HRV: heart rate variability; PCS: Pain Catastrophizing Scale; POMS 2: Profile of Mood State 2; ROM: range of motion; SF-12: Short-Form Health Survey; VR: virtual reality.

Baseline parameter measurements will be conducted on study participants before the start of the intervention. Baseline measurements will be taken during 3 usual home-visit rehabilitation as (without VR). VR applied home-visit rehabilitation will be carried out once a week, for a total of 10 times. Assessments will be conducted at the baseline, the end of the first intervention, the end of the fifth intervention, and the end of the tenth intervention. Parameter measurements in the study will include pain score, heart rate variability, range of motion, and various patient questionnaires and interviews to evaluate motivation, mood state, catastrophic thoughts of pain, and impressions about VR-applied rehabilitation. For the safety assessment of VR-applied home-visit rehabilitation, the presence and extent of symptoms related to the use of VR equipment and viewing of contents will be ascertained. Data will be collected by the researcher both at baseline and during the VR sessions. The researcher is present and observes the user during the rehabilitation visit, which includes the use of VR and collects and records a range of data. This ensures that the quality of the data collected is controlled. After the end of this clinical study, patients will receive home-visit rehabilitation as usual (without VR; Figure 3).

#### Variables

The primary variable is the change in pain from the baseline (before the VR interventions) to the tenth VR intervention. As pain is a subjective symptom of the study participants, it will be subjectively assessed by the Numerical Rating Scale (NRS) on a scale of 0-10, in which 0 indicates no pain at all and 10 indicates the most intense pain experienced so far [36]. The pain as the primary variable will be measured at 3 time points per rehabilitation session: before, during, and after the rehabilitation so that changes between time points will be evaluated.

Secondary variables are heart rate variability (HRV), range of motion (ROM) in the musculoskeletal system area where the pain occurs, motivation for rehabilitation (Behavioral Regulation in Exercise Questionnaire-2 [BREQ-2]), catastrophic thoughts of pain (PCS [Pain Catastrophizing Scale]), mood state (Profile of Mood State 2 [POMS 2]), and quality of life (Short-Form Health Survey [SF-12]).

HRV represents the ability of the heart to respond to physiological and environmental stimuli. Previous studies have shown the feasibility of HRV as a psychophysiological measurement for reactivity to stress [33]. HRV will be measured at the baseline and each VR intervention and changes from the baseline to the tenth intervention will be assessed.

ROM is the range in which each joint of the body can be physiologically exercised without injury or pain. It is one of the outcomes of rehabilitation and is also used to measure the effectiveness in patients with chronic pain [37,38]. ROM of the musculoskeletal system around where the pain occurs will be measured at the baseline and each VR intervention and changes from the baseline to the tenth intervention will be assessed.

The BREQ-2 is a 19-item scale based on self-determination theory to assess behavioral regulation in exercise. BREQ-2 asks questions relating to why they engage in physical activity and exercise, and their responses are measured on a 5-point Likert-type scale ranging from 1 (not true) to 5 (very true). The scale has 5 subscales measuring 5 types of exercise regulations, that are motivation, external regulation, introjected regulation, identified regulation, and intrinsic regulation [39]. The BREQ-2 will be applied at 4 time points, which consist of baseline, first, fifth, and tenth VR interventions and changes in scores between time points will be assessed.

The PCS assesses the extent to which participants experience magnification, rumination, and helplessness in response to their pain episodes [40]. The PCS has 13 items, each scored on a 5-point Likert scale from 0 to 4 (0: not at all, 4: all the time). The PCS is reported as a total composite score (range 0-52), with higher scores indicating greater catastrophic thoughts about pain. PCS scores will be measured at 4-time points, which consist of baseline, first, fifth, and tenth VR interventions, and changes in scores between time points will be assessed.

The POMS 2 questionnaire is used to monitor mood changes, particularly participants’ psychological stress. This scale consists of subjective feelings in 6 different mood states (tension, depression, anger, fatigue, confusion, and vigor). A 5-point Likert scale, ranging from 0 (not at all) to 4 (extremely), is used for each item. The higher scores indicate more pronounced emotions [41]. POMS 2 will be applied at 4-time points, which consist, of baseline, first, fifth, and tenth VR interventions, and changes in scores between time points will be assessed.

A 12-Item SF-12 is a health-related quality of life scale [42]. SF-12 consists of multiple questions to measure eight health concepts, that are physical functioning, limiting physical health problems, bodily pain, general health, vitality, social functioning, limiting emotional problems, and mental health. Information from all 12 items is used to construct physical and mental component summary measures [43]. The SF-12 will be applied at 3 time points, that are baseline, the fifth, and tenth VR interventions, and changes in scores between time points will be assessed.

Through interviews with study participants at the tenth intervention, the effects of VR-applied home-visit rehabilitation will be evaluated, including pain alleviation, promotion of motivation, improvement in mood state and pain catastrophic thinking, and patients’ and families’ impressions.

As part of the safety evaluation, the presence and degree of subjective symptoms will be checked using the NRS, which is expressed on an 11-point scale from 0 to 10, for pressure pain at the site where the VR equipment is worn, muscle strain from the neck to the shoulders due to the load of the VR equipment, and discomfort caused by viewing VR images (VR sickness) [35]. The adverse event NRS will be measured at each VR intervention.

Baseline measurements will be taken during 3 usual home-visit rehabilitation as (without VR). For pain and heart rate variability, the average of 3 rehabilitation visits on different days will be used as the baseline value. The joint range of motion and each questionnaire score will be measured once as a baseline.

The observation and examination items will be collected according to the schedule (Table 1).

**Table 1:** Schedule of the observation and examination.

	Baseline (home-visit rehabilitation)				Intervention period (VR applied home-visit rehabilitation)									
	1st	2nd	3rd	1st		2nd	3rd	4th	5th	6th	7th	8th	9th	10th
Obtaining consent	✓													
Participant background	✓													
Pain	✓	✓	✓	✓		✓	✓	✓	✓	✓	✓	✓	✓	✓
HRV^a^	✓	✓	✓	✓		✓	✓	✓	✓	✓	✓	✓	✓	✓
ROM^b^	✓			✓		✓	✓	✓	✓	✓	✓	✓	✓	✓
BREQ-2^c^	✓			✓		✓	✓	✓	✓	✓	✓	✓	✓	✓
PCS^d^	✓			✓					✓					✓
POMS 2^e^	✓			✓					✓					✓
SF-12^f^	✓								✓					✓
Interview														✓
VR^g^ adverse events				✓		✓	✓	✓	✓	✓	✓	✓	✓	✓

^a^HRV: heart rate variability.

^b^ROM: range of motion.

^c^BREQ-2: Behavioral Regulation in Exercise Questionnaire-2.

^d^PCS: Pain Catastrophizing Scale.

^e^POMS 2: Profile of Mood State 2.

^f^SF-12: Short-Form Health Survey.

^g^VR: virtual reality.

### Safety Evaluation

As part of the safety evaluation, the presence and degree of subjective symptoms will be checked for pressure pain at the site where the VR equipment is worn, muscle strain from the neck to the shoulders due to the load of the VR equipment, and discomfort caused by viewing VR images (VR sickness) [35]. Based on previous studies with patients at home, and also to measure the physical burden of wearing the head-mounted display, these items are measured using the NRS [44]. The adverse event will be measured at each VR intervention.

The condition of study participants will be observed before the start of VR use to check and assess for any problems in using VR; a researcher will be present at all times during the home visit rehabilitation, including the use of VR, to observe any changes in condition. If any participant develops adverse symptoms such as VR sickness at any stage before completing the VR session, the study will be terminated, and the participant will be excluded from the study. The study does not have a follow-up period after the VR sessions for all study participants; however, any patients who have adverse symptoms during the VR sessions will be monitored until their condition has recovered and stabilized.

### Statistical Analysis

The analysis will include patients who participate in at least one VR-applied home-visit rehabilitation. The primary variable will be analyzed for the participants who completed the baseline and all 10 interventions. Baseline characteristics will be assessed using descriptive statistics. For both the primary and secondary variables, descriptive statistics (mean, SD) will be calculated for a single group and evaluated at a 2-sided 5% level of significance, with 1-way repeated measures ANOVA in the case of data with a 2-sided distribution and Friedman test for data without a normal distribution. Relevant qualitative data will be summarized and reported as appropriate. The data are analyzed in R version 4.3.2 (R Foundation for Statistical Computing).

### Ethical Considerations

The study was approved by the Clinical Research Ethics Review Committee of Mie University Hospital (H2021-143) and registered as a clinical trial in “UMIN; University Hospital Medical Information Network” (UMIN000045039). All study participants were informed of the details of the study and gave face-to-face informed consent before the study began. Participants’ data will be anonymized and managed so that personal information cannot be identified.

## Results

Recruitment of participants began on February 22, 2022, and data collection is ongoing as of November 2024. The research results will be published in international peer-reviewed journals and through presentations at national and international conferences.

## Discussion

### Principal Findings

This study will lead to the development of new rehabilitation-based solutions for homebound patients who have difficulty obtaining adequate relief from chronic pain. A rehabilitation program with VR has been reported to alleviate chronic pain [21], and a mechanism of this alleviation is to distract patients from the pain by immersion in VR [19]. However, there is currently insufficient clinical evidence regarding the effects of VR for chronic pain. Especially in home-visit rehabilitation, the feasibility of using VR over the medium to long term and its effectiveness has not been fully clarified.

Existing chronic pain management measures, such as pharmacotherapy, intervention therapy, and psychotherapy, may be difficult to manage adequately because of the risk of side symptoms and the physical and psychosocial burden on the patient [16,17]. Therefore, there is a need for nonpharmacological chronic pain management measures that can be easily implemented at home.

In the current study, exercise-induced pain will be alleviated by using VR during home-visit rehabilitation, thereby increasing ROM. As increased ROM enhances the effectiveness of rehabilitation [45], chronic pain will be improved through rehabilitation. Furthermore, since pain relief with VR alleviates psychophysiological stress conditions [19], HRV should show a state of parasympathetic dominance in the activity of the autonomic nervous system [33]. The improvements in pain will also promote motivation, pain catastrophizing thinking, and mood state, measured by BREQ-2, PCS, and POMS 2, respectively [39-41], even in homebound patients who are difficult to motivate and have poor psychosocial health [9,10]. The experience of (simulated) natural landscapes brought about by VR will improve psychosocial factors in homebound patients, who infrequently leave their homes, and help improve quality of life in the medium to long term measured by SF-12 [42,43].

Therefore, this study will contribute to the development of novel rehabilitation-based solutions for homebound patients who have had difficulty obtaining adequate relief from chronic pain.

Although VR is generally considered to be a safe and noninvasive procedure with few side effects, the use of VR has been reported to induce VR sickness [35]. One must be aware of the potential occurrence of VR sickness and carefully observe the patient’s condition during the administration of VR. It is assumed that many patients at home are older adults, which may be a barrier to the introduction of VR into home rehabilitation. For VR to be successfully applied to older people, careful induction sessions are important, and the process needs to be evaluated. Limitations of this feasibility study are the small number of cases as well as the lack of blinding and controls.

### Conclusion

Future validation studies will consider conducting randomized controlled trials as clinical trials to verify efficacy.

## Abbreviations

BREQ-2: Behavioral Regulation in Exercise Questionnaire-2
HRV: heart rate variability
NRS: Numerical Rating Scale
PCS: Pain Catastrophizing Scale
POMS 2: Profile of Mood States 2nd Edition
ROM: range of motion
SF-12: Short-Form Health Survey
VR: virtual reality

## Data Availability

The datasets generated or analyzed during this study will not be publicly available but will be available from the corresponding author on reasonable request.
